# SAA1 Has Potential as a Prognostic Biomarker Correlated with Cell Proliferation, Migration, and an Indicator for Immune Infiltration of Tumor Microenvironment in Clear Cell Renal Cell Carcinoma

**DOI:** 10.3390/ijms24087505

**Published:** 2023-04-19

**Authors:** Zhijie Xu, Yunfei Wu, Guanghou Fu, Xiaoyi Chen, Junjie Sun, Junjie Tian, Peng Jiang, Yimin Wang, Baiye Jin

**Affiliations:** 1 Department of Urology, School of Medicine, The First Affiliated Hospital, Zhejiang University, Hangzhou 310009, China; 2 Zhejiang Engineering Research Center for Urinary Bladder Carcinoma Innovation Diagnosis and Treatment, Hangzhou 310024, China

**Keywords:** SAA1, tumor microenvironment, immune infiltration, mast cells resting, patient survival, prognostic marker, PDL1, clear cell renal cell carcinoma (ccRCC)

## Abstract

The tumor microenvironment (TME) plays an important part in the initiation and development of clear cell renal cell carcinoma (ccRCC). However, an understanding of the immune infiltration in TME is still unknown. Our study aims to explore the correlation between the TME and the clinical features, as well as the prognosis of ccRCC. In the present study, ESTIMATE and CIBERSORT computational methods were applied to calculate the proportion of tumor-infiltrating immune cells (TICs) and the amount of immune and stromal fractions in the ccRCC form The Cancer Genome Atlas (TCGA) database. Then, we sought to find out those immune cell types and genes which may play a significant role and validated them in the GEO database. Furthermore, an immunohistochemical analysis of our external validation dataset was used to detect SAA1 and PDL1 expression in the ccRCC cancer tissues and corresponding normal tissues. Statistical analysis was performed to study the relationship between SAA1 and clinical characteristics, as well as PDL1 expression. Furthermore, a ccRCC cell model with SAA1 knockdown was constructed, which was used for cell proliferation and the migration test. The intersection analysis of the univariate COX and PPI analysis were performed to imply Serum Amyloid A1 (SAA1) as a predictive factor. The expression of SAA1 was significantly negatively correlated to OS and positively correlated to the clinical TMN stage system. The genes in the high-expression SAA1 group were basically enriched in immune-related activities. The proportion of mast cells resting was negatively correlated with SAA1 expression, indicating that SAA1 may be involved in the maintenance of the immune status for the TME. Moreover, the PDL1 expression was positively related to the SAA1 expression and negatively correlated with the patients’ prognosis. Further experiments revealed that the knockdown of SAA1 inhibited ccRCC development through suppressing cell proliferation and migration. SAA1 may be a novel marker for the prognosis prediction of ccRCC patients and may play a vital role in the TME by mast cell resting and PDL1 expression. SAA1 has the potential to become a therapeutic target and indicator for immune target therapy in ccRCC treatment.

## 1. Introduction

According to the statistics of GLOBOCAN 2020, approximately 431,288 people were newly diagnosed as having renal cell carcinoma (RCC) and about 179,368 people died from RCC in 2020 [1]. RCC is one of the most frequent cancers originating from the urinary system, with occult onset, easy chemo-resistance, and a limited therapeutic effect of radiotherapy. Furthermore, 70–80% of RCC is clear cell renal cell carcinoma, which is characterized by robust lipid and glycogen accumulation [2]. Despite the recent progress in tumor treatment, surgical resection remains the preferred curative therapy for these patients. With the discovery of new molecular targets, new chemotherapy medications such as sunitinib, which targets vascular endothelial growth factor receptors (VEGFRs) and platelet-derived growth factor receptors (PDGFRs), often function as the standard first-line therapy for advanced ccRCC [3]. In addition, the immune checkpoint inhibitors are used to improve patients’ antitumor immune response by regulating the activity of immune cells. The combination of tyrosine kinase inhibitors (TKIs) such as pazopanib and immune checkpoint inhibitors (ICIs), such as pembrolizumab has played a beneficial role in the treatment of advanced ccRCC [4]. Motezer et al. [5] reported that the combination of ICIs and ipilimumab (anti-CTLA-4) plus nivolumab (anti-PD-1) had a better overall survival (OS) and objective response rates than sunitinib only for advanced RCC patients.

In the literature, more evidence has emerged to support that the tumor microenvironment (TME), which refers to the structure of the tumor tissue including important components such as stromal cells, vascular cells, and immune cells, exerts a critical influence on tumor progression and metastasis. The association between the TME and tumor’s biological behavior (such as unlimited proliferation, resisting apoptosis, accelerating cell cycle, and evading immune surveillance) has been established in recent years, and the TME has been revealed to significantly affect patients’ therapeutic responses and overall survival in lung cancer [6] and prostate cancer [7]. The TME mainly consists of resident stromal cells and recruited immune cells. Stromal cells are indispensable in tumors and TME, which affect metastasis initiation and extracellular matrix remodeling [8]. Recently, more and more studies pay attention to the effect of immune cells (macrophages, neutrophils, dendritic cells, innate lymphoid cells, myeloid-derived suppressor cells, natural killer cells, T cells, and B cells) in the TME on tumor initiation and progression [9,10]. The tumor infiltrating lymphocyte (TIL) was predictive of the response, and, therefore, is a promising indicator for therapeutic effects in RCC [11]. TIL was also closely connected with the overall survival of ccRCC and a low lymphocyte fraction in the tumor indicated a poor outcome of advanced ccRCC. This relevance supported the immune therapy of RCC, such as PD-1 and PD-L1 inhibitors, for metastatic RCC [12,13]. Recently, Zhou et al. reported that HHLA2 and PD-L1 co-expression predicts poor prognosis in patients with ccRCC, which is also significantly associated with TILs and suggests that the PFS and OS of tumor patients can be influenced by the tumor immune microenvironment [14]. Cai et al. showed that the simultaneous repression of mTOR and the inhibition of PD-L1–mediated signaling enhanced the CD8+ cytolytic function and obstructed tumor growth in a xenografted model of human RCC in nude mice [15]. Therefore, we presume that the adaptive immune response involved with the TME is closed related with the stage of RCC. In addition, it is a challenge to perform appropriate genetic analysis which could precisely reflect the dynamic regulation of the immune and stromal components in the TME.

With the development of transcriptome-sequence analysis and functional genomics analysis, the roles of different types of cells in the TME have been discovered. Yoshihara et al. developed ESTIMATE and CIBERSORT computational methods, which can estimate the relative proportion of the stromal and immune cells by calculating the ESTIMATEScore as well as StromalScore and ImmuneScore [16]. The study also indicated that, in RCC patients, the stromal and immune components was close related with the clinical outcome of RCC [16]. To further confirm this and identify new predictive biomarkers, we carried out this research based on The Cancer Genome Atlas (TCGA) database and GEO datasets and found the Serum amyloid A1 (SAA1). As a major acute-phase apolipoprotein reactant, SAA1 was mainly in the liver and responsible for responding to infection, potential injuries, and malignancy [17]. SAA1 has also been revealed to facilitate the production of various inflammatory mediators, cell adhesion molecules, chemokines, matrix metalloproteinases, pro-angiogenic molecules and reactive oxygen species (ROS), and pro-angiogenic molecules in some types of cells such as endothelial cells and leukocytes [18,19,20,21,22,23]. Furthermore, SAA1 has been revealed to recruit various leukocyte subsets in the TME, such as neutrophils, monocytes leukocytes, polymorphonuclear leukocytes, dendritic cells, and T lymphocyte [20,24,25,26]. These studies suggested that SAA1 might be involved in formation in the TME. Compared with immune cells and stromal cells in ccRCC samples in our research, we obtained the differentially expressed genes (DEGs) using bioinformatic methods and revealed that SAA1 has the potential to be an indicator for the alternation of the TME status in ccRCC.

## 2. Results

### 2.1. Data Acquisition and Analysis Workflow of This Study

**Figure 1:**
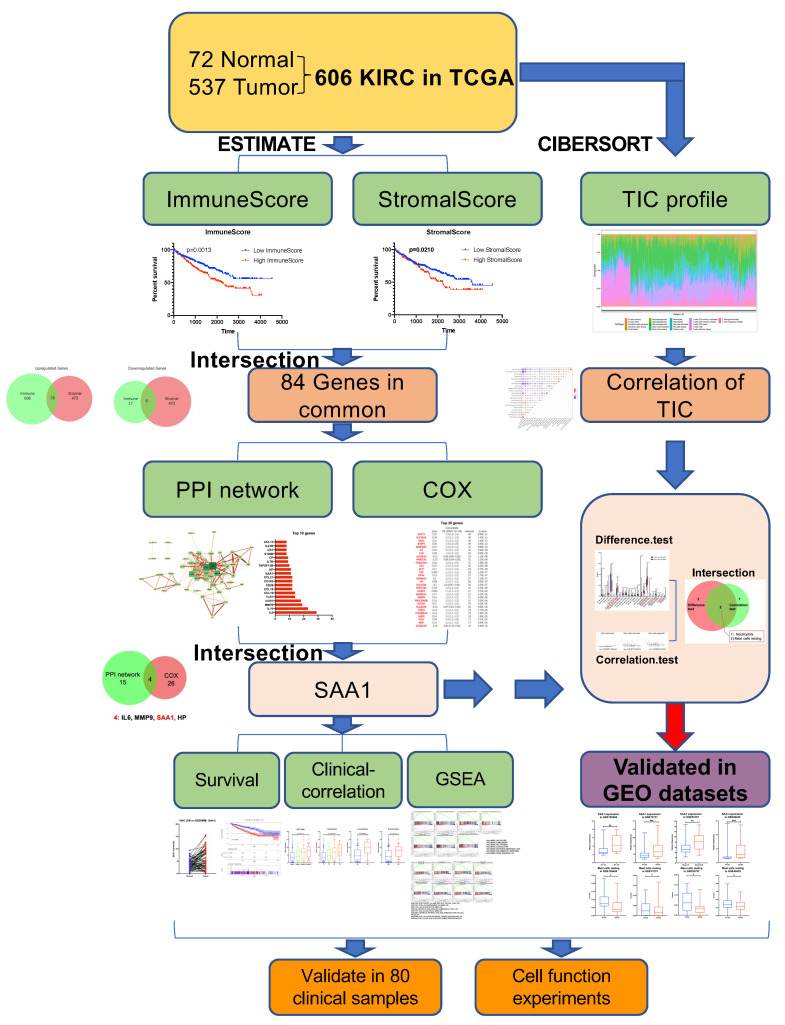
Exhibition of study workflow. TCGA ccRCC data were used in the study and were performed by ESTIMATE and CIBERSORT analysis. Out of the 84 genes in common with ImmuneScore and StromalScore, we found SAA1 was closely related with the survival and clinical features and can be an indicator for immune infiltration of TME. Furthermore, our findings were validated in GEO datasets.

Gene expression profiles and corresponding clinical information (age, gender, survival, pathological stage, tumor stage, and metastases) of all 537 (346 males and 191 females, 360 alive and 177 dead) ccRCC patients were downloaded from the TCGA Data Portal (portal.gdc.cancer.gov/, accessed on 1 June 2022). Furthermore, gene expression data and clinical information (Fuhrman grade or tumor stage) were extracted from the GEO dataset (GSE150404, GSE73731, GSE53757, and GSE40435). The workflow of our study is shown in Figure 1. In brief, the RNA-seq data of the 537 KIRC samples were calculated by ESTIMATE and CIBERSORT algorithms. The shared DEGs according to ImmuneScore and StromalScore were selected to develop the protein–protein interaction (PPI) network and univariate COX regression analysis. After this, we used the central nodes in the PPI network and the most significant factors achieved from the univariate COX regression analysis to perform intersection analysis. IL6, MMP9, SAA1, and HP were obtained from the intersection analysis, and SAA1 was selected to be the target molecule for the next series of analysis, including correlation with overall survival and clinicopathological features, COX regression, GSEA, and TICs correlation analysis. Finally, our results were verified in the GEO datasets.

### 2.2. ImmuneScore, StromalScore, and ESTIMATEScore Are Significantly Associated with Survival and Clinical Features of ccRCC

**Figure 2:**
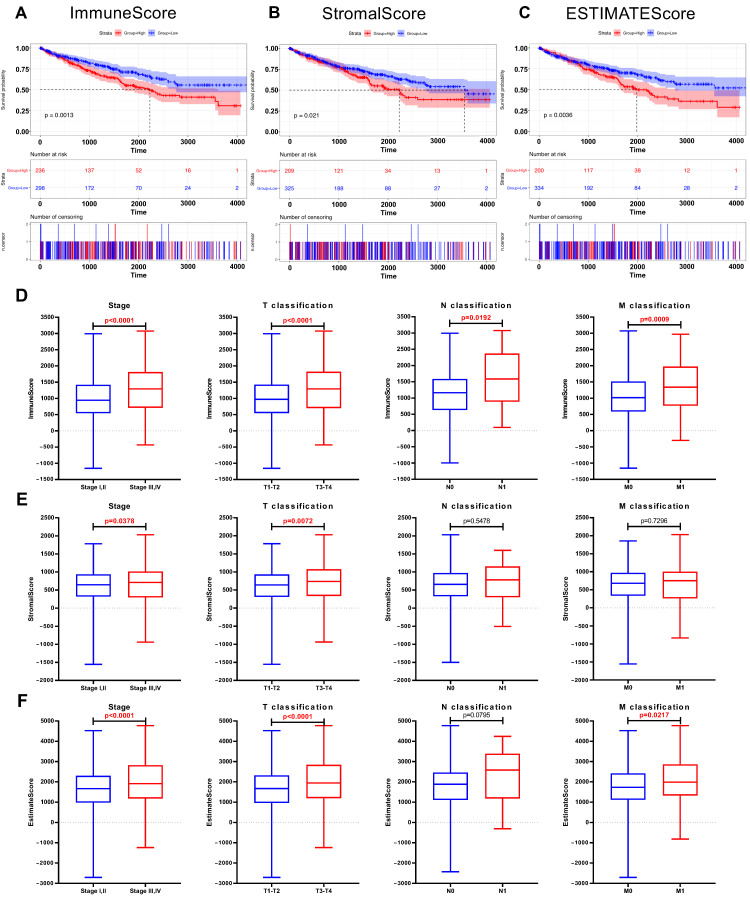
The correlation between scores and the clinicopathological features of KIRC patients. (**A**) Kaplan–Meier survival analysis for ccRCC patients stratified by ImmuneScore into high or low score (*p* = 0.001). (**B**) Kaplan–Meier survival curve for KIRC patients with high StromalScore and low StromalScore (*p* = 0.021). (**C**) Survival analysis for ccRCC patients stratified by ESTIMATEScore (*p* = 0.004). (**D**) Distribution of ImmuneScore in subgroups (stage I, II vs. stage III, IV; T1, 2 vs. T3, 4; N0 vs. N1; and M0 vs. M1). The *p* < 0.0001, < 0.0001, 0.0192, and 0.0009, respectively, by Wilcoxon rank sum test. (**E**) Distribution of StromalScore in subgroups of stage, T classification, N classification, and M classification (*p* = 0.0378, 0.0072, 0.5478, and 0.7296, respectively, by Wilcoxon rank sum test). (**F**) Comparation of ESTIMATEScore between different stage (*p* < 0.0001), T classification (*p* < 0.0001), N classification (*p* = 0.0795), and M classification (*p* = 0.0217).

The ImmuneScore, StromalScore, and ESTIMATEScore were calculated using the ESTIMATE algorithm by the “estimate” R package. All 537 samples were divided into high-score and low-score subgroups. A high ImmuneScore meant more immune cells in the TME, while a high StromalScore indicated a larger proportion of stromal cells. Kaplan-Meier survival plots indicated that the patients with higher proportions of ImmuneScores, StromalScores, and ESTIMATEScores showed significantly worse survival probabilities (*p* < 0.05, Figure 2A–C). Next, the relationship between the immune and stromal scores with the clinicopathological characteristics of the 537 ccRCC patients from TCGA was analyzed. As shown in Figure 2D–F, the ImmuneScore showed a positive correlation with the tumor stage, T classification, N classification, and M classification (Figure 2D, *p* < 0.0001, *p* < 0.0001, *p* = 0.0192, *p* = 0.0009); patients with a late tumor stage and higher T classification had a higher StromalScore (Figure 2E, *p* = 0.0378, *p* = 0.0072). In addition, higher tumor stage, T classification, and M classification patients showed increased ESTIMATEScores compared with a lower tumor stage, T classification, and M classification (Figure 2F, *p* < 0.0001, *p* < 0.0001, *p* = 0.0217). These results revealed that the progress of ccRCC was closely related to the proportion of immune and stromal components, which may affect the tumorigenesis.

### 2.3. The Common DEGs of ImmuneScore and StromalScore Were Mainly Regarded as Immune-Related Genes

**Figure 3:**
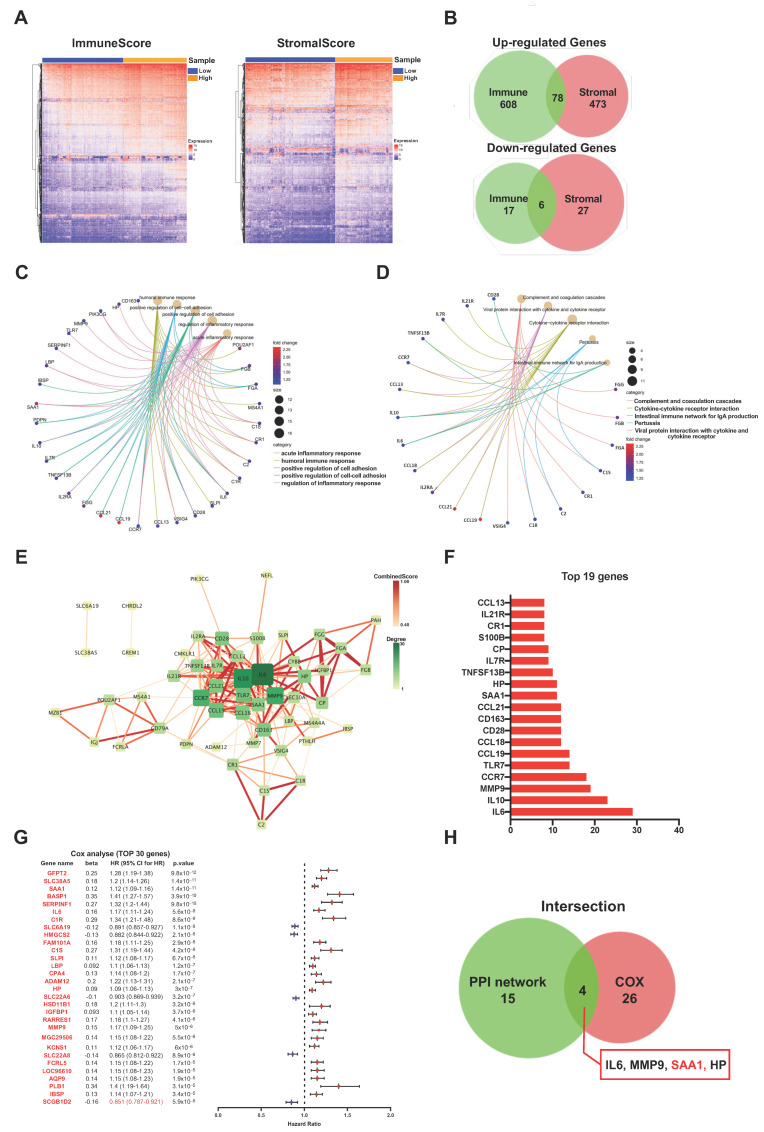
Screening of DEGs (Differentially expressed genes), GO, KEGG, and protein–protein interaction (PPI) enrichment analysis. (**A**) Heatmap showing the expression levels of DEGs between the high-score group and low-score group by ImmuneScore and StromalScore. (DEGs were determined by calculating the adjusted *p* < 0.05 and |log_2_FC| > 1 as the thresholds). (**B**) Venn plots demonstrated a total of 84 DEGs (78 up-regulated DEGs and 6 down-regulated DEGs) shared by ImmuneScore and StromalScore (*p* < 0.05 and |log_2_FC| > 1). (**C**,**D**) GO and KEGG pathways enrichment analysis for the 84 DEGs; both *p* value and q value < 0.05 were considered to be significantly enriched. (**E**) PPI network constructed with the nodes with interaction confidence value > 0.60. (**F**) The 19 genes with the most connected nodes in the network. (**G**) Top 30 significant genes with univariate COX regression analysis with 84 DEGs (*p* < 0.0001). (**H**) Venn plot depicting the 4 overlap genes (IL6, MMP9, SAA1, and HP) between the top 19 nodes in PPI and the top 30 significant genes correlated with prognosis.

To identify the genes which correlated with the immune and stromal components, a comparison analysis was performed between the high ImmuneScore/StromalScore and low ImmuneScore/StromalScore subgroups. First, the ccRCC patients were divided into high-score or low-score subgroups depending on the value according to the optimum ROC curve achieved for predicting the 5-year patient OS in regard to the ImmuneScore and StromalScore, respectively. Next, a total of 709 differentially expressed genes (686 up-regulated and 23 down-regulated genes) were identified between the high ImmuneScore and low ImmuneScore groups (Figure 3A,B). Similarly, for StromalScore, our results identified 551 over-expressed genes and 33 down-regulated genes (Figure 3A,B). Subsequently, using intersection analysis to filter the overlap genes, the Veen plot showed that 78 genes were overexpressed in both high ImmuneScore and high StromalScore patients while 6 genes were both down-regulated in ccRCC patients shared by a low score. We hypothesized that these differentially expressed genes (DEGs) (a total of 84 genes) may play key roles in the regulation of TME. Furthermore, gene ontology (GO) enrichment analysis was performed, and the result showed that these DEGs were closely related with the immune-related GO terms, such as acute inflammatory response, humoral immune response, and regulation of inflammatory response (Figure 3C). Likewise, the Kyoto Encyclopedia of Genes and Genomes (KEGG) enrichment analysis revealed that these DEGs were mainly enriched in the cytokine-cytokine receptor interaction, the intestinal immune network for IgA production, and the viral protein interaction with cytokine and the cytokine receptor (Figure 3D). These results suggested that the main functions of DEGs were correlated with immune-related activities, which indicated that an important feature of the TME in ccRCC was the involvement of the immune system.

### 2.4. PPI Network Analysis, Univariate Cox Regression of 84 DEGs

The STRING tool was used to explore the protein interactions in depth. The interplay among the above 84 genes was displayed in Figure 3E, and the bar plots displayed the top 19 genes of the DEGs by the number of interaction nodes (Figure 3F). To enrich for DEGs related to prognosis prediction, we performed univariate COX regression analysis among these 84 DEGs (Figure 3G). Next, the overlap of the genes was identified among the top 19 hub genes ranked by the number of interaction nodes of the PPI network and the top 30 factor genes related to prognosis ordered by the *p* value of univariate COX regression. Consequently, four overlap genes (IL6, MMP9, SSA1, and HP) were identified (Figure 3H).

### 2.5. The Correlation of SAA1 with Survival and TNM Stages of ccRCC Patients

**Figure 4:**
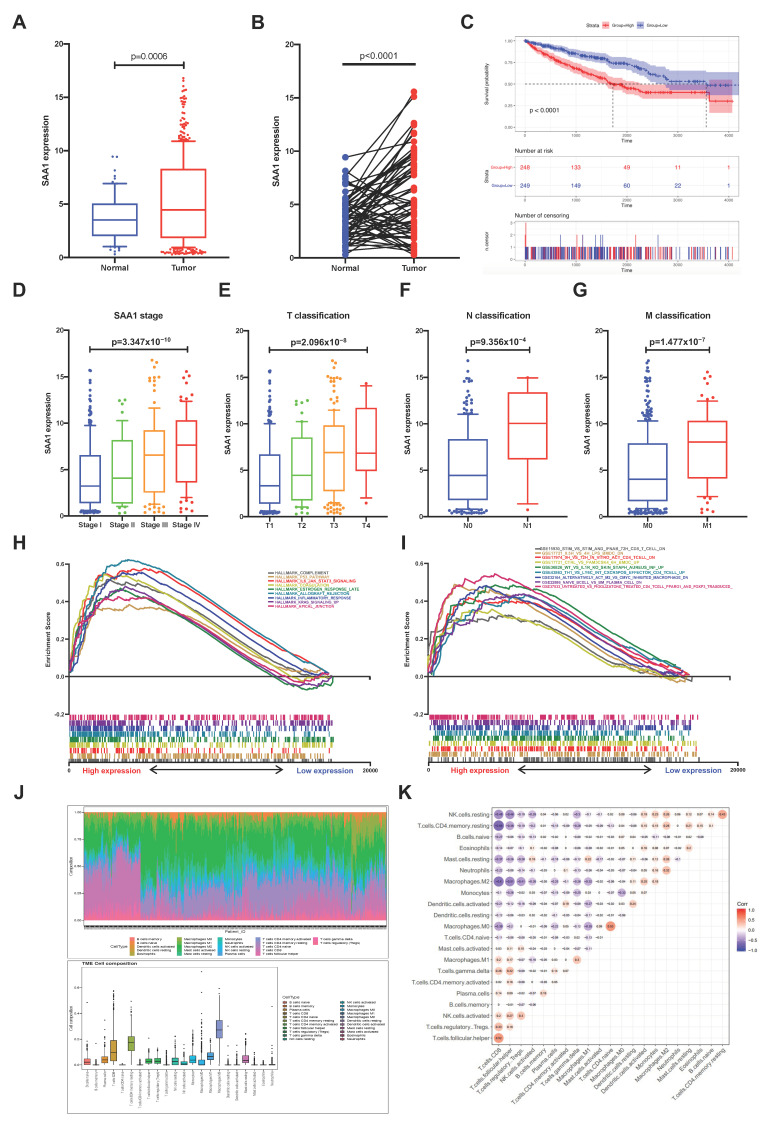
Correlation between survival and clinicopathological features of KIRC patients, GSEA, and TIC analysis. (**A**) Differential expression analysis of SAA1 between adjacent normal and ccRCC samples. (**B**) The differential expression analysis of SAA1 in matched normal-tumor pairs (*p* < 0.0001). (**C**) Kaplan-Meier survival analysis for ccRCC patients with different SAA1 expression, *p* < 0.0001 by log-rank test. (**D**–**G**) The expression of SAA1 was positively correlated with tumor stage, T stage, N stage, and M stage. (**H**) GSEA results showed significant enrichment of immune-related signaling pathways in HALLMARK collection by high SAA1 expression ccRCC patients. (**I**) GESA results revealed enriched immunologic gene sets in C7 collection by high SAA1 expression KIRC patients. (**J**) Bar plot showed the proportion of each of the 21 kinds of TICs in ccRCC tumor sample. and TME cell composition in ccRCC tumor samples. (**K**) Correlation heatmap showing the correlation between the 21 kinds of TICs.

In order to explore the potential relationship between SAA1 and clinical features of ccRCC patients, all KIRC samples were divided into a SAA1 high-expression group and SAA1 low-expression group (the SAA1 median expression was used as a cutoff value). The survival analysis revealed that the ccRCC patients with SAA1 high expression had the worse survival rate (Figure 4C). Furthermore, the analysis to evaluate the relationship with SAA1 and clinical characteristics was conducted (Supplemental Table S1), and the Wilcoxon rank sum test showed that the expression of SAA1 was significantly upregulated in the tumor samples compared with the normal samples (Figure 4A). A paired analysis of the SAA1 expression in normal tissue and tumor tissue of the same patients revealed similar results (Figure 4B). These results clearly revealed the positive correlation between SAA expression in the TME and ccRCC patients’ outcomes. In particular, the level of SAA1 expression increased with the progression of TNM staging in ccRCC patients (Figure 4D–G).

### 2.6. SAA1 Can Be an Indicator of TME Status for ccRCC Patients

Our results suggested that SAA1 were positively associated with the outcome and TNM classification of ccRCC patients; the comparison between the high-expression samples and the median level of SAA1 expression samples was conducted using GSEA, as well as the low-expression samples. Furthermore, Figure 4H and Supplemental Table S2 showed that the genes in the high SAA1 expression group were principally related to immune-related activities, such as complement, allograft rejection, and inflammatory response. The GSEA of the high SAA1 expression group using the C7 collection from MSigDB showed a statistically significant enrichment of immune-related gene sets such as the immunologic gene sets and multiple immune functional gene sets (Figure 4I and Supplemental Table S2). These results indicated that SAA1 might be valuable as a potential indicator to estimate the status of the TME in ccRCC patients.

### 2.7. Correlation between SAA1 Expression and Proportion of TICs

**Figure 5:**
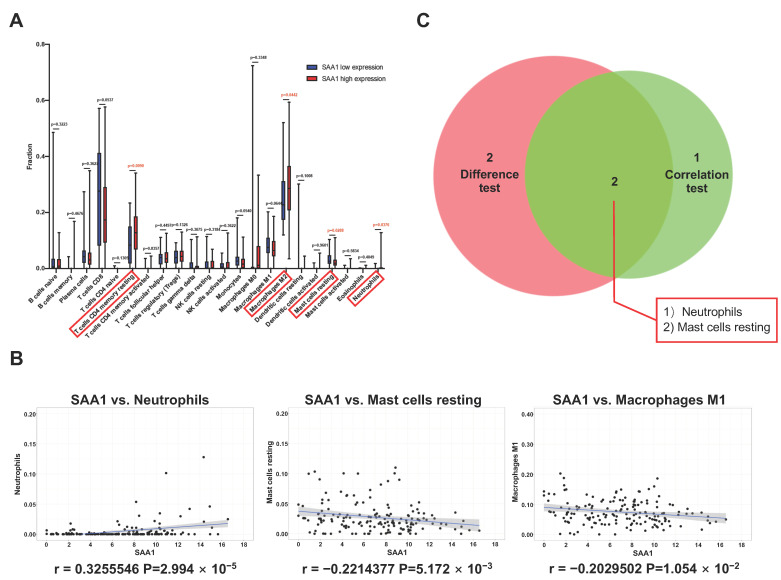
Correlation between TICs fraction and SAA1 expression in ccRCC patients. (**A**) The violin plot showed the abundance differences of 21 kinds of immune cells between SAA1-high group and SAA1-low group in KIRC tumor samples. (**B**) Scatter plot showed that 3 kinds of TICs proportion were significantly correlated with the SAA1 expression (*p* < 0.05). (**C**) Venn plot result revealed that neutrophils and mast cells resting were significantly correlated with SAA1 expression by difference and correlation tests.

To better understand the correlation between the tumor immune microenvironment and SAA1 expression, the CIBERSORT analysis was applied to estimate the abundance of tumor-infiltrating immune subsets and construct immune cell profiles in the KIRC samples covering 21 kinds of major immune cell populations (Figure 4J,K). The results indicated that a total of four types of TICs showed significantly differences between the SAA1 low expression group and SAA1 high expression group (Figure 5A). The correlation analysis showed that three kinds of TICs were close related with the expression of SAA1: neutrophils were positively correlated with SAA1 expression and mast cells resting and macrophages M1 were negatively corelated with SAA1 expression (Figure 5B). Taken together, neutrophils and mast cells resting both passed the difference and correlation tests and possessed statistical significance (Figure 5C).

### 2.8. Proportion of Mast Cells Resting Was Significantly Associated with Favorable OS, Stage, and Metastases of ccRCC

**Figure 6:**
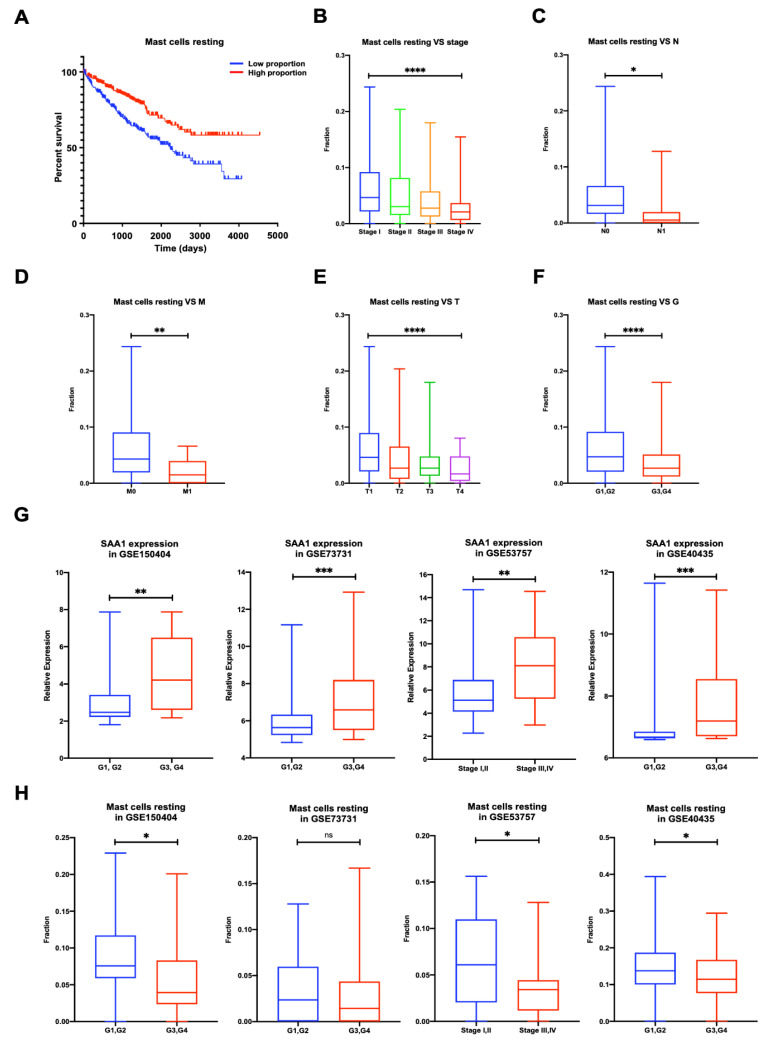
Proportion of mast cells resting was significantly negatively correlated to OS, stage, TNM stage, and grade of ccRCC, and validation of the correlation between SAA1 expression, mast cells resting proportion, and tumor grade or tumor stage in validation GEO cohorts. (**A**) Less mast cells resting predicted poorer OS; (**B**) less mast cells resting was associated with higher stage; (**C**) less mast cells resting was associated with higher chances of lymph node metastases; (**D**) less mast cells resting was associated with metastases. (**E**) Less mast cells resting was associated with higher T stage. (**F**) Less mast cells resting was associated with higher Fuhrman grade. ccRCC, clear cell renal cell carcinoma; OS, overall survival. (**G**) Correlation of SAA1 expression and tumor grade or stage in GEO datasets. (**H**) Correlation of mast cells resting and tumor grade or stage in GEO datasets. Note: * *p* < 0.05, ** *p* < 0.01, *** *p* < 0.001, **** *p* < 0.0001, ns *p* > 0.05.

To validate whether immune cells play a role in promoting ccRCC patients’ progression, we applied the CIBERSORT method and analyzed the relationship between neutrophils, mast cells resting, and patients’ OS, stage, and metastases. The results showed that the proportion of mast cells resting was significantly negatively correlated with ccRCC patients’ overall survival (Figure 6A), stage (Figure 6B), N stage (Figure 6C), M stage (Figure 6D), T stage (Figure 6E), and grade (Figure 6F).

### 2.9. Validation of the Correlation between SAA1 Expression, Mast Cells Resting Proportion, and Tumor Grade or Tumor Stage in Validation GEO Cohort

To validate the relationship between the SAA1 expression, mast cells resting proportion, and tumor grade or stage, we verified the SAA1 expression and mast cells resting proportion in the GEO datasets by different tumor grades or tumor stages (Figure 6G,H). The expression of SAA1 in the low grade (G1 and 2) or low stage (stage I and II) was significantly lower than the high grade (G3 and 4) or high stage (stage III and IV). However, the high grade (G3 and 4) or high stage (stage III and IV) had a lower fraction of mast cells resting than the low grade (G1 and 2) or low stage (stage I and II).

### 2.10. Validation of SAA1 Was Up-Regulated in ccRCC, Positively Correlated with PDL1 Expression, and Predicted Poor Therapeutic Effect

**Figure 7:**
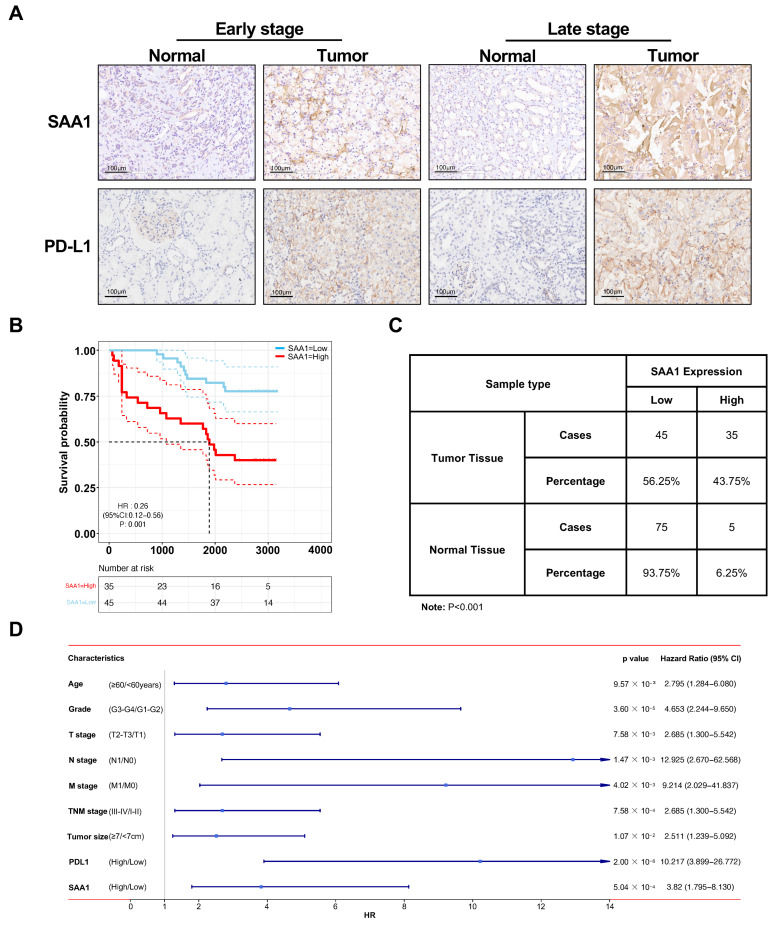
Validation of SAA1 in ccRCC in external independent cohort. (**A**) Immunohistochemistry staining of SAA1 in early stage and late stage ccRCC. (**B**) OS rates stratified by low SAA1 expression and high SAA1 expression from the TCGA database. The median was used as the dividing line for high or low SAA1 expression. ccRCC patients with high expression of SAA1 had a worse OS rate than those with low expression of SAA1. (**C**) SAA1 expression in ccRCC tissues and adjacent normal tissues (*n* = 80). (**D**) Cox regression analyses of each clinical feature, PD-L1, SAA1, and patient survival in our clinical validation dataset.

In order to further validate the role of SAA1 in the development and progression of ccRCC, clinical ccRCC tumor samples and adjacent normal tissues were collected for IHC staining analysis. As shown in Figure 7A,C, the expression of SAA1 was significantly higher in the ccRCC tumor tissues with advanced samples compared with early stage or normal samples. Next, we divided the 80 patients into high expression groups and low expression groups, and the relationship between SAA1 and clinical features was also assessed. Our cohort data not only revealed the overexpression of SAA1 in ccRCC but also the significant relationship between SAA1 expression and pathological grade, T infiltration, AJCC stage, and PDL1 expression (Table 1). Moreover, the negative association between SAA1 and overall survival was further elucidated by Kaplan-Meier survival analysis (Figure 7B). We also performed Cox regression to identify the correlations between each clinical feature, PD-L1, SAA1, and patient survival in our clinical validation dataset. As shown in Figure 7D, for all included clinical features (age, grade, T stage, N stage, M stage, TNM stage, tumor size), PD-L1, and SAA1 were powerful and risky (HR > 1) prognostic indicators in our dataset (Figure 7D).

### 2.11. The Clinicopathologic Features in Combination with SAA1 Can Further Improve the Prediction Accuracy of Prognosis

**Figure 8:**
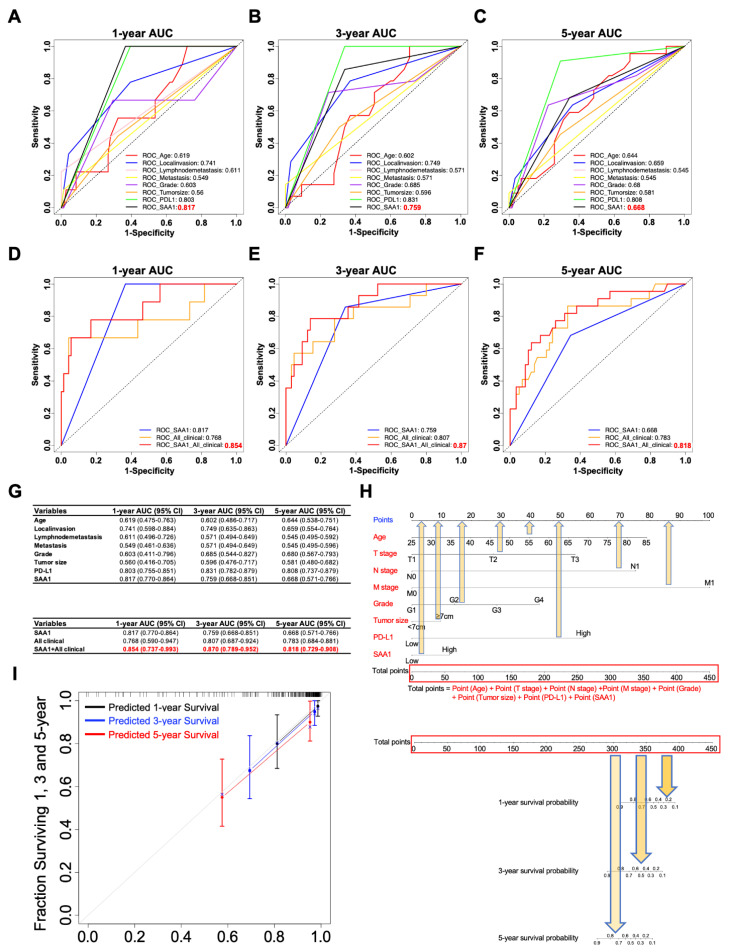
SAA1 as a potent prognostic factor in ccRCC. (**A**–**C**) Time-dependent ROC curves for assessing the predictive ability of SAA1 and clinicopathological features (Age, T stage, N stage, M stage, tumor grade, tumor size, PDL1 levels, and SAA1 levels) at different time points (**A**): 1 year, (**B**): 3 years, (**C**): 5 years in ccRCC. (**D**–**F**) Comparisons of the prognostic accuracy between SAA1, all clinicopathological features and combination of all clinicopathological features and SAA1 levels. ROC curves at different time points after diagnosis: (**D**) 1 year, (**E**) 3 years, and (**F**) 5 years. (**G**) The 95% confidence interval (CI) of AUC for each variable. (**H**) The nomograms incorporating age, T stage, N stage, M stage, Grade, Tumor size, and PD-L1 and SAA1 levels for predicting proportion of ccRCC patients with 1-year, 3-year and 5-year survival probability. (**I**) Calibration plot for analyzing the prediction ability of the nomogram between predicted and observed at 1, 3, and 5 years.

In order to verify whether SAA1 is a potential prognostic factor in ccRCC patients, we compared the prognostication performance of the above traditional clinicopathologic features (including age, T stage, N stage, M stage, grade, tumor size, and immune therapy molecular PD-L1) and our biomarker SAA1. As shown in Figure 8A–C, we found that SAA1 had the highest AUC values at 1 and 3 years than the clinical factors; however, SAA1 was inferior to PD-L1 at 3 and 5 years.

As SAA1 is a biomarker which can be detected not only in blood but also in a tumor sample, it was independent of other ccRCC clinicopathological features, which indicated that SAA1 could predict the outcome of a ccRCC patient pre-operation according to the expression of SAA1 in the biopsy or blood samples. Next, we verified whether SAA1 added prognostic value to the current often-used TNM classification system, which mostly depends on clinicopathological characteristics. The ROC curves indicated that, when SAA1 was combined with important clinicopathologic features including age, T stage, N stage, M stage, grade, and tumor size, the combination model achieved a better performance than when alone, respectively (Figure 8D–F). When combined with clinical features, SAA1 could significantly improve specificity and sensitivity across the widest range of cutoffs (AUC) for 1-, 3-, and 5-year overall survival (Figure 8G).

In order to further explore the prognostic value of SAA1, a subgroup analysis was performed by subdividing patients with different features (age: ≥60 vs. <60, grade: G3/G4 vs. G1/G2, T stage: T2/T3 vs. T1, N stage: N1 vs. N0, M stage: M1 vs. M0, TNM stage: II-IV vs. I, tumor size: ≥7 cm vs. <7 cm, PD-L1: high vs. low) into high-risk and low-risk subgroups (Supplemental Figure S1). The detailed results showed that combining SAA1 and patient clinical features exhibited excellent prognostic values. Thus, in this study, we developed a nomogram integrating both SAA1 expression level and clinical important features to more conveniently predict the overall survival in ccRCC patients who had undergone nephrectomy (Figure 8H). For example, an 80-year-old ccRCC patient with a 10 cm T3 tumor, positive lymph node, no distant metastasis, G2 tumor, high PD-L1, and high SAA1 had a total score of 310.5 (=72.5 + 55 + 75 + 28 + 10 + 57.5 + 12.5). The patient’s 3-year survival rate would be 80% and 75% at 5 years. Next, the predictive performance of the nomogram was evaluated using a calibration curve, which showed that the predicted 3- and 5-year overall survival rates were significantly correlated with the observed proportions (Figure 8I).

### 2.12. SAA1 Is Highly Expressed in ccRCC Cells and Promotes ccRCC Cell Proliferation, Migration, and Invasion

**Figure 9:**
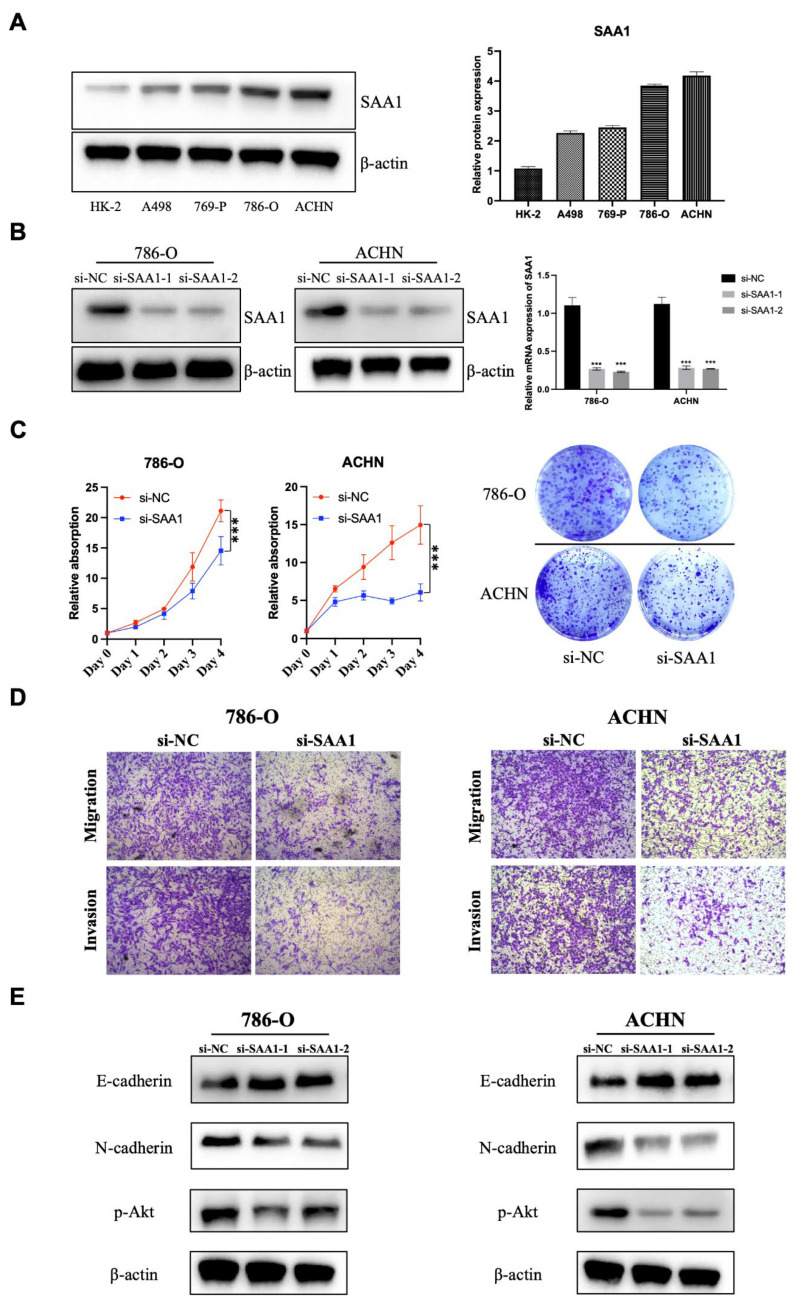
Effect of SAA1 on the proliferation and EMT of ccRCC cells. (**A**) Protein expression of SAA1 in ccRCC cell lines (A498, 769-P, 786-O, and ACHN) and normal renal epithelial cell line (HK-2). (**B**) Knock-down efficiency of si-NC, si-SAA1-1, and si-SAA1-2 was validated by Western blotting and qRT-PCR. (**C**) Cell counting kit-8 assay and colony formation assay were used to detect the proliferation ability of SAA1 knockdown ccRCC cell lines. (**D**) Transwell assay was performed to detect the migration and invasion of ccRCC cells (20×). (**E**) Effect of SAA1 knockdown on the expression of EMT-related proteins by Western blotting. Note: *** *p* < 0.001.

In the previous sections, we illustrated that SAA1 is closely related to the prognosis of ccRCC patients, which can be used as a biomarker of disease’s outcomes and new therapeutic targets. Here, we tried to explore the role of SAA1 in ccRCC. First, Western blot sand qRT-PCR results indicated that SAA1 was abnormally overexpressed in the ccRCC cells lines compared to normal renal epithelial cell HK-2 (Figure 9A). Next, the si-SAA1 and the corresponding control si-NC were transfected into ccRCC cells 786-O and ACHN, respectively. The results of qRT-PCR and Western blot showed that we had successfully knocked down SAA1 in cells 786-O and ACHN (Figure 9B). Furthermore, the CCK-8 assay showed that the knockdown of SAA1 significantly inhibited ccRCC cell proliferation (Figure 9C), and the colony formation assays revealed that the numbers of colonies decreased after SAA1 knockdown in the two cell lines (Figure 9C). Moreover, the results of the transwell assay showed that the knockdown of SAA1 inhibited the ccRCC cancer cell migration and invasion (Figure 9D). We further performed Western blot and the results showed that after the knockdown of SAA1, the EMT-related molecular (E-cadherin and N-cadherin) significantly changed and p-Akt decreased (Figure 9E). These results indicated that the knockdown of SAA1 significantly restrained the proliferation, migration, and invasion. These changes might be caused by the regulation of the Akt signal pathway.

## 3. Discussion

A solid tumor is not simply a mass of malignant tumor cells, but a complex composed of stromal and immune cells as well. Furthermore, the development of the next-generation sequencing and more and more data available in the TCGA database and GEO database has provided researchers and scientists with an unprecedented ability to study tumors [27,28]. In the present study, we aimed to recognize TME-related genes associated with the classification of TNM stages as well as survival in KIRC patients. SAA1 was identified as a potential marker involved in immune activities. Furthermore, we performed ESTIMATE and CIBERSORT analysis and the results revealed that SAA1 might be an indicator for immune infiltration of the tumor microenvironment in ccRCC patients.

TME played a significant role in the initiation and development of tumors. More and more studies have focused on exploring the potential therapeutic targets contributing to the remodeling of TME and fostering the transition of the TME from tumor-friendly to tumor-suppressed. Our results indicated that the immune infiltration in the TME contributed to a higher stage, more invasion, more chance of metastases, and poor outcomes of ccRCC. All of these results emphasized the significance of studying the interaction between the tumor component and immune component, by which much more effective treatment regimen can be developed [29,30,31,32]. As a heterogeneous tumor, ccRCC was always infiltrated with immune cells such as tumor-infiltrating lymphocytes (TILs) and, thus, would also be considered as an immunogenic cancer [33,34]. Recent trials proposed that the combination of anti-angiogenic therapy with targeted immunotherapy as one of the first-line treatments could significantly overcome the resistance by interacting with the TME [4,30]. Therefore, it is necessary to explore a novel approach for the immunotherapy of ccRCC. In our study, we performed the transcriptomic analysis of KIRC in TCGA database, which showed that the increased expression of SAA1 was statistically correlated with the advanced TNM stages and dismal outcomes. In conclusion, our results suggested that SAA1 has the potential to be not only a prognostic marker but also a therapeutic target in ccRCC. Moreover, the recent expansion of the lipid nanoparticle technology and PROTAC technology may provide the possibility of targeting SAA1 to restrain the tumorigenesis of ccRCC [35,36,37,38]. Lipid nanoparticle technology could help specifically deliver siRNA and facilitate target mRNA degradation without limited bioavailability following systemic administration. Meanwhile, PROTAC technology could lead to target protein degradation with the help of ubiquitin-protease systems. New emerging technologies offer endless possibilities for targeted therapies.

As a major acute-phase apolipoprotein reactant, SAA1 was mainly in the liver and responsible for responding to infection, potential injuries, and malignancy; it has also been discovered as overexpressed in various types of cancers and relates with a poor prognosis [17,39,40]. A study analyzed 26 serum samples collected from mRCC patients before and after sunitinib therapy and the result showed that SAA1 expression levels were positively associated with a poor response to sunitinib, which identified SAA1 as a protein marker for the prediction of tyrosine kinase inhibitors (TKIs) therapy response [41]. Our results revealed that the expression of SAA1 increased in progressive ccRCC patients, which seemed to be consistent with a series of malignancies. Several studies pointed out that the majority of SAA1 function is linked to toll-like receptors (TLRs) 2 and 4 [20,42,43]. In addition, SAA1 was also described as a ligand for additional receptors such as the G protein-coupled receptor and formyl peptide receptor 2 (FPR2), and synergized with CXCL8 to enhance neutrophil recruitment through the activation of FPR2 [20]. Thus, SAA1 might play a pivotal role in tumors and regulate the TME through two pathways: one is its chemotactic effect for several leukocyte subtypes by activating G protein-coupled receptor FPRL1/FPR2 and the other is its biological activities, such as cytokine induction, MMP-9 release, and ROS generation by mediating via TLRs. In addition, it has been reported that SAA1 might be involved in regulating macrophage polarization in the TME. Li et al. reported that after the stimulation of SAA, the polarization of U937 cells changed into M2b macrophages [44]. Similarly, Sun et al. revealed the M2 polarization of human CD14+ monocytes when treated with rSAA [45]. Therefore, we further analyzed the relationship between SAA1 expression and the TME. The GSEA results showed that immune-related signaling pathways, such as complement, allograft rejection, and inflammatory response, were significantly enriched in the SAA1 high-expression group. These results implied that SAA1 played a role in the immune response and status of the TME.

It was also well-known that SAA1 was crucial for the functions of recruiting different cell types, including various leukocyte subsets. In our present study, by applying CIBERSORT analysis for the proportion of TICs, the results demonstrated that T cells CD4 memory resting, macrophages M2, mast cells resting, and neutrophils were correlated with SAA1 expression in ccRCC patients. Among them, mast cells resting were both significantly correlated with the expression of SAA1 in ccRCC patients (*p* = 0.005) and overall survival (*p* < 0.0001). Mast cells are recognized as unique tissue-resident immune cells that secrete a diverse array of biologically active compounds which could stimulate, modulate, or suppress the immune response. A series of studies have reported that mast cells are consistently infiltrating tumors; however, their role whether as a driving or an opposite force for cancer progression is still controversial. In our study, the negative expression between the amounts of mast cells resting and SAA1 expression in ccRCC patients suggested that SAA1 might be responsible for the preservation of immune-activate status in the TME. We validate the correlation between the mast cells and SAA1 expression in the histological sections and explore how SAA1 influences the recruitment of mast cells in the further experiments.

Using ESTIMATE algorithm, we determined the TME-related genes in ccRCC through the functional enrichment analysis of ccRCC samples in the TCGA database and validated the results in the GEO datasets. SAA1 has the potential to be a prognostic factor for ccRCC patients. Moreover, SAA1 might be an indicator for the maintenance of the TME status in immune-dominant. Therefore, further investigation should be conducted to clarify the accuracy of a combined analysis of SAA1 expression, the amounts of tumor-infiltrating mast cells resting, and the types of mutation driven prior to SAA1 inhibitor treatment for ccRCC patients.

In this study, we also validated the improved prognostic value of SAA1 for 1-, 3- and 5-year survival in an independent cohort, which outperformed most clinicopathologic features, such as T stage, N stage, and M stage. Most importantly, combining the SAA1 with clinicopathologic features improves the predicted score for patient survival, suggesting that clinical data and SAA1 provided independent and complementary prognostic information. Therefore, it can be used preoperatively by analyzing tumor biopsies or tumor-derived proteins in the patient blood prior to clinical intervention. We also developed an integrated nomogram which could help improve not only preoperative but also after surgery risk classification and enhance personalized clinic decision-making. More importantly, we found that SAA1 expression was closely related with PD-L1 expression, so it can help patient decision-making for immunotherapy.

ccRCC is the most frequent pathological type of RCC, accounting for 70–80% RCC patients. The pathological types of the remaining RCC patients mainly include papillary, chromophobe, and so on. Recent research has found that the complex heterogeneity of the renal tumor was reflected in the composition of the TME. The tumor core of papillary renal cell carcinoma was mainly enriched with macrophages, while the periphery of collecting duct carcinoma was mostly rich in B cells and CD3+ T cells [46]. Although TME existed heterogeneity, one study identified the core immune-related genes (IGLL5 and IL2RA) in ccRCC and papillary renal cell carcinoma [47]. The above results enlightened us to further explore whether SAA1 could be used as an immune-related biomarker in the rarer histology of RCC.

As a TME component in RCC, although the PD-L1 expression is recognized as a predicted marker for response to immune checkpoint inhibitors (ICIs) in several cancers such as non-small cell lung carcinoma [48], it has not proven to be a relevant predictive biomarker in metastasis ccRCC. Recently, many studies (KEYNOTE 426 [4], Checkmate 214 [5] and JAVELIN RENAL 101 [49]) reported that PD-L1 expression was insufficient to predict which metastasis ccRCC patient could benefit from ICI therapy. Our risk score model in the nomogram based on clinical features and molecular markers (SAA1 and PD-L1) may be a new promising predictive marker in the new era of immune checkpoint inhibitors. The overexpression of SAA1 induced tumor proliferation and migration by regulating the Akt pathway, which might determine the clinical outcome of patients with ccRCC.

## 4. Materials and Methods

### 4.1. Data Preparation

The RNA-seq data of 537 ccRCC patients and their corresponding clinical follow-up data such as sex, age, stage, the presence or absence of metastasis, survival, and prognosis were retrieved from The Cancer Genome Atlas (TCGA, https://portal.gdc.cancer.gov/tcga/, accessed on 1 June 2022). In addition, four transcriptome RNA sequencing datasets (GSE150404, GSE73731, GSE53757 and GSE40435) and their clinical information (Fuhrman grade or tumor stage) were extracted from the GEO database (https://www.ncbi.nlm.nih.gov/geo/, accessed on 1 June 2022). Genes with FPKM = 0 in more than half of the samples were excluded, and next transformed with log2(FPKM + 1) for the subsequent analysis.

### 4.2. Calculation of ImmuneScore, StromalScore and ESTIMATEScore

ImmuneScores, StromalScores, and ESTIMATEScores were calculated using the ESTIMATE algorithm to each sample [15]. The positive correlations between these scores and the proportion of immune and stromal and the sum of immune and stromal were visualized, respectively.

### 4.3. Tumor-Infiltrating Immune Cells (TICs) Profile

The CIBERSORT method [26] was used for interfering the TIC abundance profile in the bulk tumor transcriptomes of all tumor samples; 158 tumor samples were included by quality filtering with *p* < 0.05 for the following analysis.

### 4.4. Survival Analysis

R package survival and survminer were performed for survival analysis. Kaplan-Meier curves and the log-rank test were used to distinguish clinical prognostic features using the KIRC kidney cancer cohort. *p* < 0.05 was considered significant.

### 4.5. Analysis of Differentially Expressed Genes (DEGs)

We first identified the DEGs between the high-score and low-score groups according to ImmuneScore and StromalScore, respectively. Moderated t-tests were computed using the “Limma” R package [27] in R 3.6.3 to identify DEGs among the ImmuneScore and StromalScore subgroups with log2FC (|log2 FoldChange| > 1) and FDR (false discovery rate) < 0.05. Moreover, “Complex heatmap” was used to create heatmaps of DEGs.

### 4.6. GO and Kyoto Encyclopedia of Genes and Genomes (KEGG) Enrichment Analysis

Go and KEGG enrichment analysis using the 86 DEGs were performed with R packages clusterProfiler, enrichplot, and ggplot2. Terms with *p* value and q value <0.05 were defined as significantly enriched.

### 4.7. Protein-Protein Interaction (PPI) Network Construction

The PPI network was constructed from the STRING database [28] and then reconstructed via Cytoscape of version 3.6.1. The nodes with combined scores larger than 0.4 were used for further analysis.

### 4.8. Gene Set Enrichment Analysis

Firstly, we downloaded Hallmark and C7 gene sets v7.0 collections from the Molecular Signatures Database as our target sets. Then, gene set enrichment analysis (GSEA) was performed by applying the software gsea-3.0 from https://software.broadinstitute.org/gsea/, accessed on 1 June 2022. GSEA was performed to analyze all transcriptome RNA-seq data of the 606 KIRC cases we acquired, and we defined gene sets with NOM *p* < 0.05 and FDR q < 0.05 as significant.

### 4.9. Tissue Samples and Immunohistochemistry

The tissue samples were collected from the archive of the Institute of Urology, the First Affiliated Hospital of Zhejiang University School of Medicine. All patients were asked to provide a written informed consent in compliance with the ethical regulations before enrolling in the study. Our study was carried out in according with the Declaration of Helsinki and approved by the Ethics Committee of the First Affiliated Hospital at Zhejiang University School of Medicine. Paraffin-embedded tumor samples (*n* = 80) were available from pathologically confirmed clear cell renal cell carcinoma. Immunohistochemical (IHC) staining was performed as previously described [29]. The IHC sections were incubated with anti-SAA1 (1:400; cat no. ab199030; Abcam, Cambridge, UK) and anti-PDL1 (1:250; cat no. ab213524) for 24 h at 4 °C, followed by incubating with the secondary antibody for 30 min at 37 °C. After staining with DAB and counter-staining with hematoxylin, the IHC results were examined by pathologists.

The staining percentage score of all patients were classified according to the percentage of SAA1- or PDL1-stained cancer cells’ intensity as follows: (1) a score of 0 as “Negative”; (2) a score of 1 as “1–24%”; (3) a score of 2 as “25–49%”; (4) a score of 3 as “50–74%”; and (5) a score of 4 as “75–100%”. Furthermore, the staining intensity scores of each patient were based on the following rules: (1) “Negative” received a score of 0; (2) “1+” a score of 1; (3) “2+” a score of 2; and (4) “3+” a score of 3. The patients were divided into two groups according to the total score = “staining percentage score” × “staining intensity score”. The samples with scores ≤6 were defined as the low expression group, while the samples with scores >6 were defined as the high expression group.

### 4.10. Cell Culture and Transfection

The human renal cell carcinoma cell lines (786-O and ACHN) and human renal epithelial cell (HK-2) were obtained from ATCC. These cell lines were maintained in RPMI 1640, MEM (minimum essential medium), and DMEM (Dulbecco’s Modified Eagle’s Medium) containing 10% fetal bovine serum (FBS, Thermo Fisher Scientific, Waltham, MA, USA) at 37 °C with 5% CO_2_. Si-RNA against SAA1 and its corresponding negative control (si-NC) were purchased from Sunya Biological Co., Ltd. (Hangzhou, Zhejiang), which were transfected into cells using Lipofectamine 2000 reagent.

### 4.11. Cell Counting Kit-8 (CCK-8) and Colony Formation Assay

After being transfected with si-SAA1 or si-NC, the 786-O and ACHN cells were placed in 96-well plates with 1000 cells per well at 37 °C and 5% CO2. Next, the cells were treated with 10% CCK-8 reagent for 1 h and assessed at day 0, 1, 2, 3, and 4 using the Bio-rad microplate reader at 450 nm. In addition, for colony formation assay, 5 × 102 cells were transferred into each well of 6-well plates. After incubation for 8–10 days, the cells were stained with 0.5% crystal violet and photographed.

### 4.12. Transwell Migration and Invasion

Furthermore, 786-O and ACHN cells (1 × 104) with si-SAA1 or si-NC were seeded in 100 μL of a serum-free medium in the upper chamber coated with or without Matrigel (1:20; BD Biosciences, San Jose, CA, USA). Meanwhile, the lower chamber was added with medium supplemented with 10% FBS. Next, the numbers of invaded or migrated cells were counted in five random microscopic fields under the DP70 CDD system (Olympus Corp., Tokyo, Japan).

### 4.13. Western Blot

ACHN and 786-O with different treatments were lysed with RIPA lysis buffer added to 1% of the cocktail protease inhibitor (Thermo Fisher Scientific). After concentration measurement, 15 μg/10 μL protein samples were separated by 12% Tris-acetate gels and then transferred onto the PVDF membranes. Then, the membrane was blocked in 5% non-fat milk for 1 h before incubation with the primary antibody SAA1 (1:1000; Abcam) at 4 °C overnight. The next day, the membrane was incubated with secondary antibodies for 1 h at room temperature after washing with TBST 3 times. β-actin (1:2000; Abcam) was used as an internal reference. Protein bands were detected by EZ-ECL chemiluminescence detection kit and analyzed using Image-Pro Plus software 6.0.

### 4.14. Statistics

The analyses of scores with clinicopathological characteristics were performed by R, and Wilcoxon rank sum or Kruskal-Wallis rank sum test were performed as the significance test. Kaplan-Meier plots were used to investigate the association between overall survival and gene expression. The correlation was verified by Pearson analysis. Univariate Cox regression was used to investigate the relationship between the expression level of DEGs and patient overall survival (OS). Variables that were statistically significant in the univariate analysis were included in a Cox multivariate regression survival analysis. The scatter plot was generated using Graphpad Prism8 software. *p* < 0.05 was considered statistically significant.

## Figures and Tables

**Table 1:** Relationship between SAA1 Expression and Tumor Characteristics in Patients with RCC.

Features	No. of Patients	SAA1 Expression		*p* Value ^a^
		Low	High	
All patients	80	45	35	
Age (years)				0.499
<60	40	21	19	
≥60	40	24	16	
Gender				0.194
Male	52	32	20	
Female	28	13	15	
Grade				0.017 *
1	20	9	11	
2	33	25	8	
3	26	10	16	
4	1	1	0	
T classification				0.038 *
T1	45	30	15	
T2	29	14	15	
T3	6	1	5	
Lymphatic metastasis (N)				0.188
N0	78	45	33	
N1	2	0	2	
Distant metastasis (M)				0.188
M0	78	45	33	
M1	2	0	2	
AJCC stage				0.035 *
I	45	30	15	
II	27	14	13	
III	6	1	5	
IV	2	0	2	
Tumor size				0.046 *
<7 cm	53	34	19	
≥7 cm	27	11	16	
PDL1 expression				<0.001 ***
Low	43	32	11	
High	37	13	24	

^a^ Pearson chi-square test or Fisher exact test were used for comparison between subgroups. * *p* < 0.05, *** *p* < 0.001.

## Data Availability

TCGA dataset for clear cell renal cell carcinoma. https://www.cancer.gov/about-nci/organization/ccg/research/structural-genomics/tcga/studied-cancers/renal-cell-clear-cell, accessed on 1 June 2022.

## Supplementary Materials

The supporting information can be downloaded at: https://www.mdpi.com/article/10.3390/ijms24087505/s1.

## Abbreviations

SAA1: serum amyloid A1. TME: tumor microenvironment. ccRCC: clear cell renal cell carcinoma. TIC: tumor-infiltrating immune cell. TCGA: The Cancer Genome Atlas. OS: overall survival. TIL: tumor-infiltrating lymphocyte. GEO: Gene Expression Omnibus. T: tumor stage. N: lymph node metastasis. M: distant metastasis. DEG: differentially expressed gene. KEGG: Kyoto Encyclopedia of Genes and Genomes. PPI: protein-protein interaction. VEGFR: vascular endothelial growth factor receptor. PDGFR: platelet-derived growth factor receptor. RCC: renal cell carcinoma. ROS: reactive oxygen species. TKIs: tyrosine kinase inhibitors. ICIs: immune checkpoint inhibitors. DEGs: differentially expressed genes. TILs: tumor-infiltrating lymphocytes. mTOR: mammalian target of rapamycin. TLRs: toll-like receptors. FPR2: formyl peptide receptor 2.
