# Supplementary material for: SAA1 Has Potential as a Prognostic Biomarker Correlated with Cell Proliferation, Migration, and an Indicator for Immune Infiltration of Tumor Microenvironment in Clear Cell Renal Cell Carcinoma
Source: Int J Mol Sci. 2023 Apr 19;24(8):7505. doi: 10.3390/ijms24087505 (PMC10138873; doi:10.3390/ijms24087505)
Supplement: Supplementary file 1 [file ijms-24-07505-s001.zip › SM_Captions.pdf]

SUPPLEMENTARY MATERIAL CAPTIONS

Supplementary Figure S1. Kaplan-Meier survival analysis for external validation data (N=80) based on the prognostic classifier stratified by Age, Grade, T stage, N stage, AJCC stage, M stage, Tumor size and PD-L1 levels. (A) Kaplan-Meier curves for Age, and further stratified according to SAA1 levels by Age<60 (middle panel, N = 40), Age≥60 (right panel, N = 40). (B) Kaplan-Meier curves for Grade, and further stratified according to SAA1 levels by G1-G2 (middle panel, N = 53), G3-G4 (right panel, N = 27). (C) Kaplan-Meier curves for T stage, and further stratified according to SAA1 levels by T1 (middle panel, N = 45), T2-T3 (right panel, N = 35). (D) Kaplan-Meier curves for N stage, and further stratified according to SAA1 levels by N0 (right panel, N = 78). (E) Kaplan-Meier curves for AJCC stage, and further stratified according to SAA1 levels by Stage I (middle panel, N = 45), Stage II-IV (right panel, N = 35). (F) Kaplan-Meier curves for M stage, and further stratified according to SAA1 levels by M0 (right panel, N = 78). (G) Kaplan-Meier curves for Tumor size, and further stratified according to SAA1 levels by <7cm (middle panel, N = 53), ≥7cm (right panel, N = 27). (H) Kaplan-Meier curves for PD-L1, and further stratified according to SAA1 levels by PD-L1 low (middle panel, N = 43), PD-L1 high (right panel, N = 37).The difference between the two curves were determined using two-sided log-rank test.

Supplementary Table S1. Clinicopathological characteristics statistics of KIRC patients from TCGA.

Supplementary Table S2. Enriched gene sets.
