# Supplementary material for: SAA1 Has Potential as a Prognostic Biomarker Correlated with Cell Proliferation, Migration, and an Indicator for Immune Infiltration of Tumor Microenvironment in Clear Cell Renal Cell Carcinoma
Source: Int J Mol Sci. 2023 Apr 19;24(8):7505. doi: 10.3390/ijms24087505 (PMC10138873; doi:10.3390/ijms24087505)
Supplement: Supplementary file 1 [file ijms-24-07505-s001.zip › Table S1.pdf]

**Supplementary Table S1.** Clinicopathological characteristics statistics of KIRC patients from TCGA.

| Clinical characteristics | | Total (537) | % |
|---|---|---|---|
| Age at diagnosis (y) | young age (<=60) | 266 | 49.5 |
| | old age (>60) | 271 | 50.5 |
| Gender | Male | 346 | 64.4 |
| | Female | 191 | 35.6 |
| Stage | I | 269 | 50.1 |
| | II | 57 | 10.6 |
| | III | 125 | 23.3 |
| | IV | 84 | 15.6 |
| | NA | 2 | 0.4 |
| T classification | T1 | 275 | 51.2 |
| | T2 | 69 | 12.8 |
| | T3 | 182 | 33.9 |
| | T4 | 11 | 2.0 |
| M classification | M0 | 426 | 79.3 |
| | M1 | 79 | 14.7 |
| | NA | 32 | 6.0 |
| N classification | N0 | 240 | 44.7 |
| | N1 | 17 | 3.2 |
| | NA | 280 | 52.1 |
