# Supplementary material for: SAA1 Has Potential as a Prognostic Biomarker Correlated with Cell Proliferation, Migration, and an Indicator for Immune Infiltration of Tumor Microenvironment in Clear Cell Renal Cell Carcinoma
Source: Int J Mol Sci. 2023 Apr 19;24(8):7505. doi: 10.3390/ijms24087505 (PMC10138873; doi:10.3390/ijms24087505)
Supplement: Supplementary file 1 [file ijms-24-07505-s001.zip › Table S2.pdf]

Supplementary Table S2. Enriched gene sets.

MSigDB collection	Gene set name	NES	NOM p-val	FDR q-val
h.all.v7.0.symbols.gmt				
SAA1 high expression	HALLMARK_APOPTOSIS	1.784	0.0096	0.0474
vs	HALLMARK_GLYCOLYSIS	1.732	0.0178	0.0336
SAA1 low expression	HALLMARK_COMPLEMENT	1.727	0.0041	0.0231
	HALLMARK_P53_PATHWAY	1.660	0.0098	0.0219
	HALLMARK_IL6_JAK_STAT3_SIGNALING	1.623	0.0298	0.0232
	HALLMARK_COAGULATION	1.619	0.0080	0.0204
	HALLMARK_ ESTROGEN_RESPONSE_LATE	1.607	0.0117	0.0190
	HALLMARK_ ALLOGRAFT_REJECTION	1.591	0.0451	0.0187
	HALLMARK_ INFLAMMATORY_RESPONSE	1.546	0.0490	0.0213
	HALLMARK_ KRAS_SIGNALING_UP	1.506	0.0303	0.0221
	HALLMARK_ APICAL_JUNCTION	1.490	0.0459	0.0205
c7.all.v7.0.symbols.gmt				
SAA1 high expression	GSE15930_STIM_VS_STIM_AND_IFNAB_72H_CD8_T_CELL_DN	1.828	0.0042	0.0248
vs	GSE17721_0.5H_VS_4H_LPS_BMDC_DN	1.828	0	0.0244
SAA1 low expression	GSE17974_0H_VS_72H_IN_VITRO_ACT_CD4_TCELL_DN	1.826	0.0186	0.0241
	GSE17721_CTRL_VS_PAM3CSK4_6H_BMDC_UP	1.825	0	0.0240
	GSE36826_WT_VS_IL1R_KO_SKIN_STAPH_AUREUS_INF_UP	1.824	0.0166	0.0237
	GSE43863_TH1_VS_LY6C_INT_CXCR5POS_EFFECTOR_CD4_TCELL_UP	1.824	0.0125	0.0233
	GSE32164_ALTERNATIVELY_ACT_M2_VS_CMYC_INHIBITED_MACROPHAGE_DN	1.824	0.0041	0.0230
	GSE22886_NAIVE_BCELL_VS_BM_PLASMA_CELL_DN	1.819	0.0000	0.0236
	GSE37533_UNTREATED_VS_PIOGLIZATONE_TREATED_CD4_TCELL_PPARG1_AND_FOXP3_TRASDUCED_UP	1.819	0.0020	0.0233
	GSE37532_TREG_VS_TCONV_CD4_TCELL_FROM_LN_UP	1.818	0.0084	0.0231

NES: normalized enrichment score; NOM: nominal p-value; FDR: false discovery rate. Gene sets with NOM p-value less than 0.05 and FDR q-value less than 0.05 were considered as statistical significance. Only several leading sets enriched in SAA1 high expression both in HALLMARK and C7 were listed here due to the large number of enriched gene sets.
