# Supplementary figures and images for: SAA1 Has Potential as a Prognostic Biomarker Correlated with Cell Proliferation, Migration, and an Indicator for Immune Infiltration of Tumor Microenvironment in Clear Cell Renal Cell Carcinoma
Source: Int J Mol Sci. 2023 Apr 19;24(8):7505. doi: 10.3390/ijms24087505 (PMC10138873; doi:10.3390/ijms24087505)

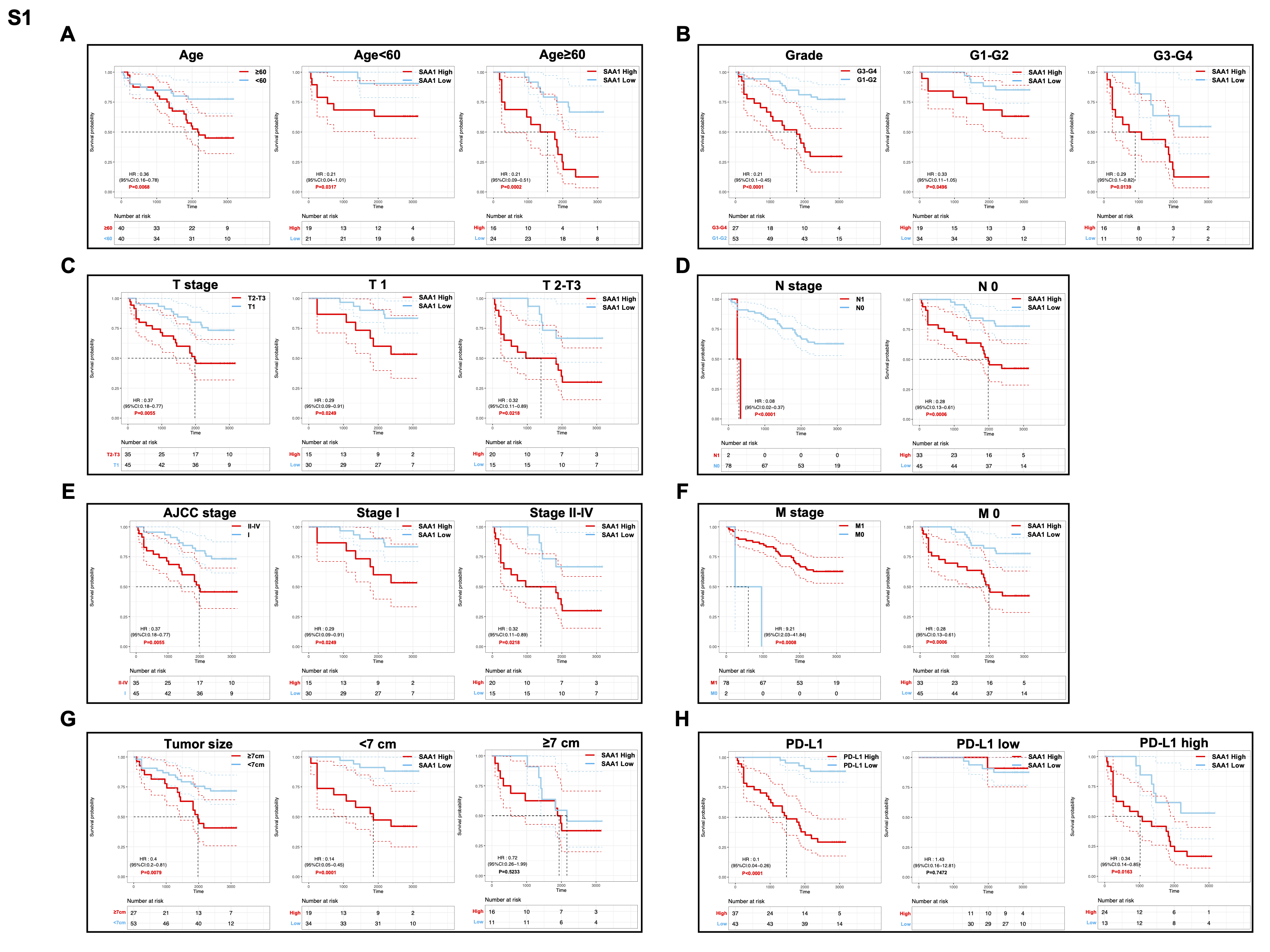

Supplement: Supplementary file 1 [file ijms-24-07505-s001.zip › Figure S1.tiff]
